# Low-Power Dynamic Object Detection and Classification With Freely Moving Event Cameras

**DOI:** 10.3389/fnins.2020.00135

**Published:** 2020-02-20

**Authors:** Bharath Ramesh, Andrés Ussa, Luca Della Vedova, Hong Yang, Garrick Orchard

**Affiliations:** ^1^Life Science Institute, The N.1 Institute for Health, National University of Singapore, Singapore, Singapore; ^2^Temasek Laboratories, National University of Singapore, Singapore, Singapore

**Keywords:** object recognition, neuromorphic vision, low-power FPGA, closed-loop control, object detection, event-based descriptor, rectangular grid, FIFO processing

## Abstract

We present the first purely event-based, energy-efficient approach for dynamic object detection and categorization with a freely moving event camera. Compared to traditional cameras, event-based object recognition systems are considerably behind in terms of accuracy and algorithmic maturity. To this end, this paper presents an event-based feature extraction method devised by accumulating local activity across the image frame and then applying principal component analysis (PCA) to the normalized neighborhood region. Subsequently, we propose a backtracking-free *k*-d tree mechanism for efficient feature matching by taking advantage of the low-dimensionality of the feature representation. Additionally, the proposed *k*-d tree mechanism allows for feature selection to obtain a lower-dimensional object representation when hardware resources are limited to implement PCA. Consequently, the proposed system can be realized on a field-programmable gate array (FPGA) device leading to high performance over resource ratio. The proposed system is tested on real-world event-based datasets for object categorization, showing superior classification performance compared to state-of-the-art algorithms. Additionally, we verified the real-time FPGA performance of the proposed object detection method, trained with limited data as opposed to deep learning methods, under a closed-loop aerial vehicle flight mode. We also compare the proposed object categorization framework to pre-trained convolutional neural networks using transfer learning and highlight the drawbacks of using frame-based sensors under dynamic camera motion. Finally, we provide critical insights about the feature extraction method and the classification parameters on the system performance, which aids in understanding the framework to suit various low-power (less than a few watts) application scenarios.

## 1. Introduction

Through these fruitful decades of computer vision research, we have taken huge strides in solving specific object recognition tasks, such as classification systems for automated assembly line inspection, hand-written character recognition in mail sorting machines, bill inspection in automated teller machines, to name a few. Despite these successful applications, generalizing object appearance, even under moderately controlled sensing environments, for robust and practical solutions for industrial challenges like robot navigation and sense-making is a major challenge. This paper focuses on the industrially relevant problem of real-time, low-power object detection using an asynchronous event-based camera (Brandli et al., 2014) with limited training data under unconstrained lighting conditions. Compared to traditional frame-based cameras, event cameras do not have a global shutter or a clock that determines its output. Instead, each pixel responds independently to temporal changes with a latency ranging from a low of tens of microseconds to a high of few milliseconds. This local sensing paradigm naturally results in a wider dynamic range (120 dB), as opposed to the usual 60 dB for frame-based cameras.

Most significantly, event cameras do not output pixel intensities, but only a spike output with a precise timestamp, also termed an event, that signifies a sufficient change in log-intensity of the pixel. As a result, event cameras require lower transmission bandwidth and consume only a few hundred mW vs. a few W by standard cameras (Posch et al., 2014). In summary, event-based cameras offer a fundamentally different perspective to visual imaging while having a strong emphasis on low-latency and low-power algorithms (Conradt et al., 2009; Ni et al., 2012; Delbruck and Lang, 2013; Kueng et al., 2016).

Despite the notable advantages of event cameras, there still remains a significant performance gap between event camera algorithms and frame-based counterparts for various vision problems. This is partly due to a requirement of totally new event-by-event processing paradigms. However, the burgeoning interest in event-based classification/detection is focused on closing the gap using deep spiking neural networks (O'Connor et al., 2013; Lee et al., 2016), something that again entails dependence on powerful hardware like its frame-based counterpart. On the other hand, a succession of frames captured at a constant rate (say 30 Hz), regardless of the scene dynamics and ego-motion, works well with controlled scene condition and camera motion. Frame-based computer vision algorithms have benefited immensely from sophisticated methodologies that reduce the computational burden by selecting and processing only informative regions/keypoints within an image (Lowe, 2004; Galleguillos et al., 2008; Vikram et al., 2012; Ramesh et al., 2017a). In addition, frame-based sensing has led to high hardware complexity, such as powerful GPU requirements for efficiently re-training and deploying state-of-the-art object detection frameworks using deep neural networks (Ren et al., 2017; Redmon and Farhadi, 2018).

Since event-based vision is relatively new, only a limited amount of work addresses object detection using these devices (Liu et al., 2016; Iacono et al., 2018; Lenz et al., 2018). Liu et al. (2016) focuses on combining a frame-based CNN detector to facilitate the event-based module. We argue that using intensity images, either reconstructed from the event stream (Scheerlinck et al., 2018) or captured simultaneously (Liu et al., 2016; Iacono et al., 2018), with deep neural networks for event-based object detection may achieve good performance with lots of training data and computing power, but they go against the idea of low-latency, low-power event-based vision. In contrast, Lenz et al. (2018) presents a practical event-based approach to face detection by looking for pairs of blinking eyes. While Lenz et al. (2018) is applicable to human faces in the presence of activity, we develop a general purpose event-based, object detection method using a simple feature representation based on local event aggregation. Thus, this paper is similar in spirit to the recently spawned ideas of generating event-based descriptors, such as histogram of averaged time surfaces (Sironi et al., 2018) and log-polar grids (Ramesh et al., 2017b, 2019b), or low-level corner detectors as features (Manderscheid et al., 2019). Moreover, the proposed object detection and categorization method was accommodated on FPGA to demonstrate energy-efficient low-power vision.

**Figure 1:**
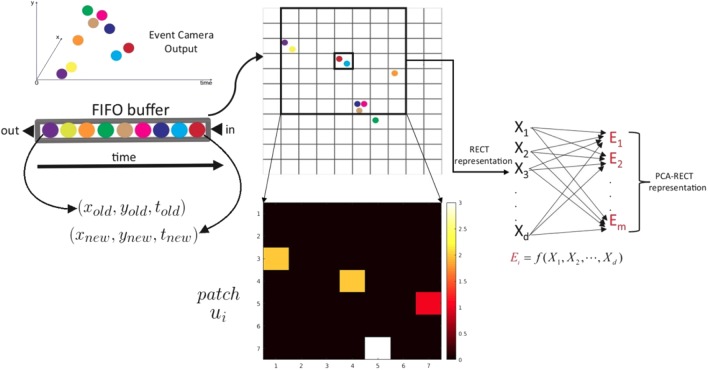
PCA-RECT representation (best viewed on monitor). Useful events are sub-sampled and filtered after applying nearest-neighbor temporal filtering and refractory filtering, termed as rectangular event context transform (RECT). The sparser RECT event representation is updated dynamically using a first in, first out (FIFO) buffer. Subsequent feature extraction is carried out by applying principal component analysis (PCA) to project RECT onto a lower-dimensional subspace to obtain the final PCA-RECT feature representation.

In contrast to the above works, this paper introduces a simple, energy-efficient approach for object detection and categorization. Figure 1  illustrates the local event-based feature extraction pipeline that is used for classification using a codebook-based method. Accordingly, efficient feature matching with the codebook is required, which is handled by a backtracking-free branch-and-bound *k*-d tree. This proposed system was ported to a field programmable gate array (FPGA) with certain critical design decisions, one of which demanded a virtual dimensionality reduction method based on the *k*-d tree, to accommodate very low-power computational needs.

This paper is an extended version of the work initially published in ACCV Workshops 2018 (Ramesh et al., 2019a). Novel contributions over (Ramesh et al., 2019a) include closed-loop aerial flight, tested for the first time using event-based sensors to the best of our knowledge, and robustness analysis using hand-held experiments (section 3.3) with critical insights into the system performance for various hyper-parameters (section 3.1.1). Additionally, this work includes a comprehensive comparison to deep learning methods (section 3.4) and further provides full implementation details in section 2, including a free-running mode implementation capable of a classification output at any point in time, as opposed to a classifier periodically operating on a set of events like (Ramesh et al., 2019a).

## 2. Materials and Methods

We follow the event-based classification framework proposed in Ramesh et al. (2017b), with the following crucial changes: a new descriptor (PCA-RECT), a virtual dimensionality reduction technique using *k*-d trees (vPCA) and a simplified feature matching mechanism to account for hardware limitations. The framework consists of four main stages: feature extraction, feature matching with a codebook, creating an object representation, which is lastly fed to a linear classifier. Additionally, we incorporate an object detector in the framework as explained in the following subsections.

### 2.1. PCA-RECT

Each incoming event, ${\text{e}}_{i}={\left(x_{i},y_{i},t_{i},p_{i}\right)}^{T}$ with pixel location *x*_*i*_ and *y*_*i*_, timestamp *t*_*i*_, polarity *p*_*i*_, is encoded as a feature vector **x**_*i*_. To deal with hardware-level noise from the event camera, two main steps are used: (1) nearest neighbor filtering and (2) refractory filtering. We define a spatial Euclidean distance between events as,

(1) $$\begin{array}{l} D_{i,j}=\|\left(\begin{array}{c} x_{i}\\ y_{i} \end{array}\right)-\left(\begin{array}{c} x_{j}\\ y_{j} \end{array}\right)\|\;. \end{array}$$

Using the above distance measure, for any event we can define a set of previous events within a spatial neighborhood, *N*(**e**_*i*_, γ) = {**e**_*i*_ | *j* < *i, D*_*i, j*_ < γ}, where $\gamma =\sqrt{2}$ for an eight-connected pixel neighborhood. When the time difference between the current event and the most recent neighboring event is less than a threshold, Θ_*noise*_, the filter can be written as

(2) $$\begin{array}{l} F_{noise}\left(e\right)=\{e_{i}|\;N(e_{i},\;\sqrt{2})\backslash N(e_{i},\;0)\;∋\;e_{j}\;|\;t_{i}-t_{j}<\Theta_{noise}\}\;. \end{array}$$

When the neighborhood is only the current pixel, γ = 0, the set of events getting through the refractory filter *F*_*ref*_ are those such that,

(3) $$\begin{array}{l} F_{ref}\left(e\right)=\left\{e_{j}|t_{i}-t_{j}>\Theta_{ref}\forall j|e_{j}\in N\left(e_{j},0\right)\right\}\;. \end{array}$$

Cascading the filters, we can write the filtered incoming events as,

(4) $$\begin{array}{l} \left\{\hat{\text{e}}\right\}=F_{noise}\left(F_{ref}\left(e\right)\right)\;. \end{array}$$

As shown in Figure 1, the incoming events ${\hat{\text{e}}}_{i}$ are first pushed into a FIFO buffer. The FIFO queue is then used to update an event-count matrix *C* ∈ ℝ^*m* × *n*^, where *m* and *n* denote the number of rows and columns of the event camera output.

(5) $$\begin{array}{l} C\left(x_{i},y_{i}\right)=C\left(x_{i},y_{i}\right)+1\;. \end{array}$$

Before pushing the latest event, the FIFO buffer of size S is popped to make space and simultaneously update the count matrix C,

(6) $$\begin{array}{l} C\left(x_{i-s},y_{i-s}\right)=C\left(x_{i-s},y_{i-s}\right)-1\;. \end{array}$$

The event-count *C* is pooled to build local representations, which are further aggregated to obtain the RECT representation of each event. In particular, let *A* be a *p* × *q* rectangular grid filter, the 2-D convolution is defined as,

(7) $$\begin{array}{l} R\left(j,k\right)=\underset{p}{\sum }\underset{q}{\sum }A\left(p,q\right)C\left(j-p+1,k-q+1\right)\;, \end{array}$$

where *p* run over all values that lead to legal subscripts of *A*(*p, q*) and *C*(*j* − *p* + 1, *k* − *q* + 1). In this work, we consider a filter containing equal weights (commonly known as an averaging filter) for simplicity, while it is worth exploring Gaussian-type filters that can suppress noisy events. The resultant 2-D representation is termed as filtered matrix *R* ∈ ℝ^(*m*/*p*) × (*n*/*p*)^, where the filter dimensions are chosen to be give integer values for *m*/*p* and *n*/*q* or conversely *C* is zero-padded sufficiently. Subsequently, the RECT representation for ${\hat{\text{e}}}_{i}$ is obtained as a patch **u**_*i*_ (see Figure 1) of dimension *d* centered at *R*(*y*/*p, x*/*q*). Subsequently, the filtered event-count patch is projected on-to a lower-dimensional subspace using principal component analysis (PCA) for eliminating noisy dimensions and improving classifier accuracy. For the sake of completion, the details of extracting the principal components (PCs) are given below.

For a set of *n* mean-centered feature vectors ${\text{u}}_{i}\in {\mathbb{R}}^{d}$ with *N*_*i*_ samples in the subset *D*_*i*_ belonging to class ω_*i*_, (*i* = 1, ⋯ , *C*), principal component analysis (PCA) seeks a projection *W* that minimizes the error function:

(8) $$\begin{array}{l} J_{PCA}\left(W\right)=\overset{N}{\underset{k=1}{\sum }}||u_{k}-v_{k}|{|}^{2}\;. \end{array}$$

where **v**_*k*_ is obtained after projection of **u**_*k*_ by W as ${\text{v}}_{k}=WW^{T}{\text{u}}_{k}$. The minimization is equivalent to finding the eigenvectors of the total scatter matrix defined as:

(9) $$\begin{array}{l} S_{T}=\overset{N}{\underset{k=1}{\sum }}\left(u_{k}-\mu \right){\left(u_{k}-\mu \right)}^{T}\;. \end{array}$$

where μ is the mean of all training samples:

(10) $$\begin{array}{l} \mu =\frac{1}{N}\overset{N}{\underset{k=1}{\sum }}u_{k}\;. \end{array}$$

The columns of *W* associated with non-trivial eigenvalues are the PCs and those with negligible eigenvalues are regarded as arising from noise. After projecting the RECT representation using the PCs, each filtered event is thus encoded as a feature vector ${\text{x}}_{i}\in {\mathbb{R}}^{d^{\prime }}$ where *d*′ < *d*.

### 2.2. Feature Selection and Matching Using *K*-d Trees

The PCA-RECT feature representation for each event is matched to a codebook for creating the object representation. However, exhaustive search is too costly for nearest neighbor matching with a codebook, and approximate algorithms can be orders of magnitude faster than exact search, while almost achieving par accuracy.

In the vision community, *k*-d tree nearest-neighbor search is popular (Silpa-Anan and Hartley, 2008; Muja and Lowe, 2009), as a means of searching for feature vectors in a large training database. Given *n* feature vectors ${\text{x}}_{i}\in {\mathbb{R}}^{d^{\prime }}$, the *k*-d tree construction algorithm recursively partitions the *d*′-dimensional Euclidean space into hyper-rectangles along the dimension of maximum variance. However, for high dimensional data, backtracking through the tree to find the optimal solution still takes a lot of time.

Research in the vision community has therefore aimed at increasing the probability of success while keeping backtracking within reasonable limits. Two similar and successfully applied approximated search methods are the best-bin-first search (Beis and Lowe, 1997) and priority search (Arya and Mount, 1993). Backtracking is accomplished in such methods by maintaining an estimate of the distance from the query point to any of the nodes down all of the branches. In the best-bin-first search, a parameter specifies the number of data points that can be checked before terminating and returning the closest point traversed up to that point. This process however still requires the computationally expensive Euclidean distance calculation to a subset of the data points in the codebook or training database.

This paper proposes a simple, backtracking-free branch-and-bound search for matching (Algorithm 1), taking advantage of the low-dimensionality of the PCA-RECT representation. The hypothesis is that, in general, the point recovered from the leaf node is a good approximation to the nearest neighbor in low-dimensional spaces, and performance degrades rapidly with increase in dimensionality, as inferred from the intermediate results in Beis and Lowe (1997). In other words, with (log_2_ *n*) − 1 scalar comparisons, nearest neighbor matching is accomplished without an explicit distance calculation. While the PCA-RECT representation is useful for software implementations, an extra PCA projection step can be computationally demanding on FPGA devices. To this end, we propose a virtual PCA-RECT representation based on the *k*-d tree, termed as vPCA-RECT.

**Algorithm 1:** KDSEARCHFAST(node, **y**): Fast *k*-d tree search for PCA-RECT

**Input**: The root of the *k*dtree structure, and a query point **y** **Output**: *k*dtree leaf node data-point *j*
1: **if** *node*.type ≠ *leaf* **then**
2: **if** **y**(*node*.splitDimension) ≤ *node*.splitThreshold **then**
3: *node* ← *node*.left
4: KDSEARCHFAST(node, **y**)
5: **else**
6: *node* ← *node*.right
7: KDSEARCHFAST(node, **y**)
8: **end** **if**
9: **else**
10: **return** *node*.dataIndex
11: **end** **if**

#### 2.2.1. vPCA-RECT

A key insight is that only a fraction of the data dimensions are used to partition the *k*-d tree, especially when the codebook size is only a few times more than the feature dimension. Therefore, instead of using the PCA-RECT representation, an alternative dimensionality reduction scheme can be implemented by discarding the unused dimensions in the *k*-d tree structure. In other words, the RECT representation is first used to build a *k*-d tree that selects the important dimensions (projection π), which are then utilized for codebook learning and classification. It is worth noting that exactly the same *k*-d tree will be obtained if the RECT data is first projected by π onto a subspace that is aligned with the coordinate axes. Since no actual projection takes place, we refer to this as a virtual projection—the irrelevant dimensions chosen by the *k*-d tree are discarded to obtain a lower-dimensional feature representation.

### 2.3. Event-Based Object Categorization and Detection

*The learning stage*: Using the PCA-RECT event representation, the learning process corresponds to creating a set of *K* features denoted as *M* = {1, 2, ⋯ , *K*} to form the codebook (also termed as dictionary). The clustering is done by randomly selecting a sufficiently large pool of event representations of various categories from the training samples. It is worth noting that the dictionary features are learned from the training set using unsupervised clustering for all the objects jointly.

In contrast to the above single-shot learning for PCA, the vPCA-RECT representation requires two codebook learning steps. First, using the RECT representation, an initial codebook of size *K*_*v*_, where *K*_*v*_ < < *K*, is built to spot the unused feature dimensions in the *k*-d tree. The unused dimensions simply correspond to those which were not used by the *k*-d tree construction algorithm to recursively partition the d'-dimensional Euclidean space into hyper-rectangles. Subsequently, the projection π is used to obtain a lower-dimensional representation for the training data and then the final codebook of size *K* is generated. The initial, smaller codebook helps to partition the RECT feature space with much higher entropy, and thus is an essential step for the virtual PCA-RECT representation.

The learning stage for detection builds on top of the categorization module, in such a way that the learning process corresponds to selecting a subset of features from the codebook for each object. In contrast to the learning phase of the categorization module, the detector features are selected from the whole training set in a supervised one-vs-all manner.

We propose to evaluate the balanced matches $Y_{+}^{k}$ to each codeword *f*_*k*_ from the target events against the matches $Y_{-}^{k}$ for all the other events to the respective feature. Mathematically, the ratio

(11) $$\begin{array}{l} D\left(k\right)=\frac{\beta_{+}^{k}Y_{+}^{k}}{\beta_{-}^{k}Y_{-}^{k}}\;,\;\text{where }\beta_{+}^{k}=\frac{\left|Y_{+}^{k}\right|}{\overset{K}{\underset{k=1}{\sum }}|Y_{+}^{k}|},\;\text{and }\beta_{-}^{k}=\frac{\left|Y_{-}^{k}\right|}{\overset{K}{\underset{k=1}{\sum }}|Y_{-}^{k}|}\;, \end{array}$$

is to be maximized. The balancing component $\beta_{+}^{k}$ denotes the percentage of target events matched to the codeword *f*_*k*_. Similarly, $\beta_{-}^{k}$ denotes the percentage of non-target events matched to the codeword *f*_*k*_. Thus, choosing the detector features with the D–largest ratios completes the learning phase.

*The classification/detection stage*: At runtime, the event representations are propagated through the *k*-d tree. On the one hand, the distribution of the codewords are then extracted and further passed to a simple linear classifier (we experimented with both linear SVM and Kernel Methods). On the other hand, the event representations propagated through the *k*-d tree are matched with the detector features. Those matched events are used to update a location map for the target object and the region with the highest activation is considered to be the final detection result.

### 2.4. FPGA Implementation

**Figure 2:**
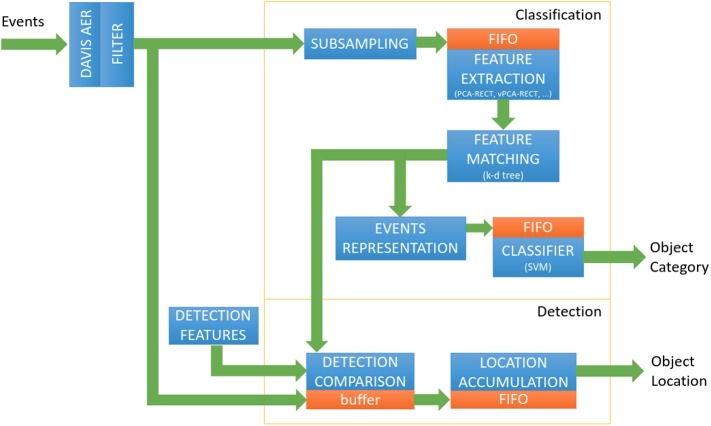
Pipeline of the proposed system.

An overview of the whole system is shown in Figure 2. Learning is performed in software first and the relevant data is transferred to the FPGA, which corresponds to the SVM weights and the information regarding the nodes of the k-d tree. This process does not require fine tuning of the FPGA code, except for the case of a different codebook size, in which only the new size has to be updated in the FPGA side. The memory initialization for the block memories is automated from the files generated during training in software.

#### 2.4.1. Categorization Pipeline

In order to showcase energy-efficient event-based object recognition, the FPGA implementation of the algorithm is designed as a series of four independent hardware units: event sub-sampling, vPCA-RECT generation, a recursive *k*-d tree and a SVM classifier output on an event-by-event basis, each of which has an independent block design. Generally, these hardware counterparts are not a direct application of the algorithm presented in the earlier section, i.e., certain design decisions were taken for this task, among them, to desist the use of an extra PCA projection along the pipeline.

**Figure 3:**
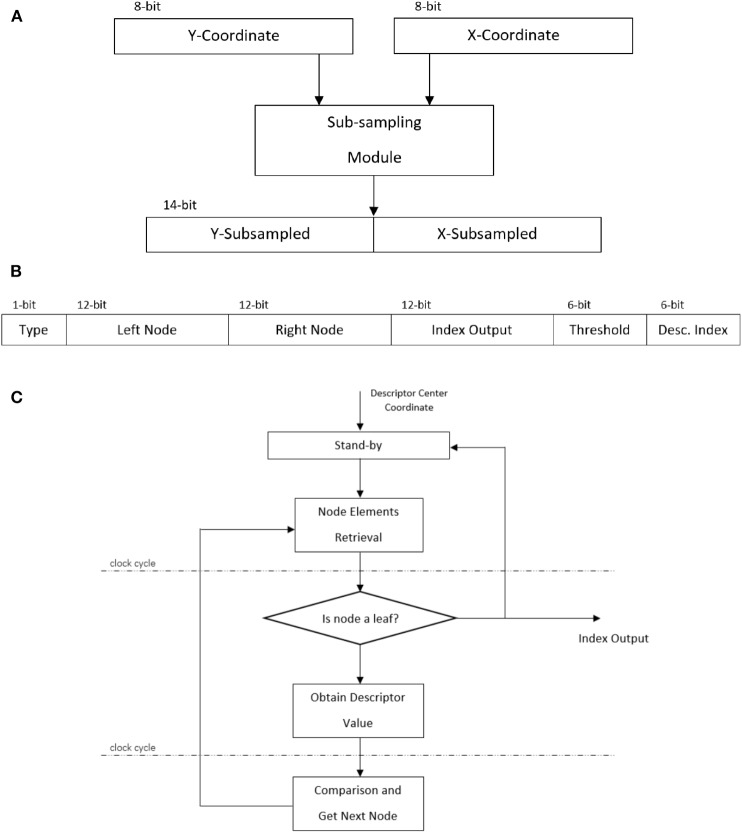
FPGA implementation details: **(A)** Sub-sampling module, **(B)** A *k*-d tree node in hardware, and **(C)** Recursive logic-driven *k*-d tree implemented in hardware.

The sub-sampling block receives the filtered event locations as input values *x* and *y*, each 8-bit in size, which are used to update the zero-padded count matrix *C* ∈ ℝ^*m* × *n*^ (Equation 5, 6). The sub-sampling behavior can be achieved in hardware through a combinatorial module that performs the division by shifting the inputs by one bit, and subsequently adding *p* and *q* to that value to obtain the sub-sampled representation (Equation 7). This results in two 7-bit values which are then concatenated to output a single memory address (Figure 3A).

The next block uses the cell-count matrix *R* ∈ ℝ^(*m*/*p*) × (*n*/*q*)^, created by a block of distributed RAM of depth ((*m*/*p*) × (*n*/*q*)) and *log*(*s*)-bits width, corresponding to the FIFO buffer size *s*, initialized to zero for generating the vPCA-RECT representation. To generate a descriptor with respect to the last event received would add a considerable overhead, since each element of the descriptor would have to be read sequentially from the block RAM while being stored by the next module. Instead, the address corresponding to the center of the descriptor is provided, i.e., the input address of the count matrix is passed over to the *k*-d tree module. This allows to trigger the *k*-d tree in one clock cycle once the count matrix is updated and later read the descriptor values based on this single coordinate. However, a new issue arises, the count matrix then can not be modified while the *k*-d tree exploration is being performed. Hence a buffering element is added between the sub-sampling and count matrix modules that will only provide the next address once there is a valid output from the tree.

The *k*-d tree nodes are represented in a 49-bit number stored in a previously initialized single port ROM of depth equal to the number of nodes. This number is conformed by the elements of a node: type, left node, right node, index output, split value and split dimension; these are concatenated and their width is shown in Figure 3B.

The *k*-d tree module follows a three steps cycle (Figure 3C). The split dimension of a *k*-d tree node provides the address that needs to be read from the cell-count matrix block RAM to get the relevant descriptor value. Next, the descriptor value is compared to the previously stored split value from the node, taking a path down the tree, left or right, depending on the boolean condition. The corresponding node to get is then retrieved from the respective left or right address element acquired in the retrieval step. This cycle repeats until the node type belongs to a leaf, then the leaf node output is made available for the classifier module. It is worth mentioning that in the software implementation of this algorithm, once the descriptor is formed, it is then normalized before being passed to the *k*-d tree. A normalization step in hardware would add a big overhead to the pipeline, disturbing its throughput, and it was removed from the FPGA implementation after verifying that the overall performance was not affected harshly. The “distribution of the codewords” normalization that is input to the SVM is an important step (Equation 12). It is implicitly performed by limiting the number of events quantized to that of the FIFO buffer size. This is more important than normalizing the descriptors. Using the N-SOD, we noticed only a small drop in test accuracy (from 98 to 93%) without normalization, which is acceptable for real-time applications.

At runtime in a software implementation, the classification is performed by a linear combination of the weights and a feature vector created by the *k*-d tree after a buffer time of *S* events. To achieve this in a hardware implementation, the depth of the feature vector would have to be transversed while performing several multiplications which would require a considerable amount of multiplier elements from the FPGA, and would affect the speed of the module. Thus, it was desired to avoid this solution and the following was proposed.

The elements of the linear combination mentioned would be acquired as readily available and would be added to an overall sum vector of length equal to the number of classes to classify, hence performing the dot product operation as one addition per event. Then, after *S*_*c*_ events, a resulting vector is formed, which is equal to the result of the same linear combination first mentioned in the software implementation. Thus, the final module to perform the classification receives the output index from the *k*-d tree and adds its corresponding classifier parameter to a sum vector of length equal to the number of classes. In parallel, this index value is stored in another FIFO element. When the queue is full, the oldest value would be passed to the module to be subtracted from the sum. This allows to have a classification output at any point in time, corresponding to the last *S*_*c*_ events.

Let *k*_*i*_ be the output of the *k*-d tree, which also corresponds to the codeword index of the dictionary, and *k*_*old*_ be the *k*-d tree index corresponding to the oldest event being removed from the FIFO buffer. Let the SVM weights and bias be $W_{SVM}\in {\mathbb{R}}^{K\times C}$ and $B_{SVM}\in {\mathbb{R}}^{1\times C}$, respectively for C object categories with K features. Thus, the former element *k*_*i*_ contributes to the SVM representation whereas *k*_*old*_ must be removed from the SVM representation. A classification output for an event *i* is computed as $S_{i}^{o}=W_{SVM}\cdot H_{i}+B_{SVM}$, considering a dictionary representation denoted by $H_{i}\in {\mathbb{R}}^{K\times 1}$. The equivalent free-running SVM update can be represented using Equation (12).

(12) $$\begin{array}{l} S_{i}^{o}=S_{i-1}^{o}+W_{SVM}\left(k_{i},1:C\right)-W_{SVM}\left(k_{old},1:C\right) \end{array}$$

Note that the number of input events used to form the feature representation *H*_*i*_ is always constant, and corresponds to the last S events that creates the PCA-RECT representation using a FIFO (Equation 6). Hence, it is not a single event that is being classified. As the queue is updated on an event-by-event basis, the classification output corresponds to the entire block, although the classification output is triggered by every event.

#### 2.4.2. Detection Pipeline

Parallel to the modules performing the classification pipeline, the aim of the detection process is to find the coordinates corresponding to “landmarks” with the highest activation after *S*_*d*_ events, and then find the most probable location for the object. Again, the algorithm was divided into multiple coherent hardware modules that would produce the same results as the original software version. The designed blocks are: landmarks detector, detection heat map and mean calculation.

First, the codewords corresponding to the landmarks that were calculated offline are loaded into a binary memory block. This module receives as input the codeword index provided by the *k*-d tree for the current event. If the feature is found as one of the landmarks, the respective event coordinates *x* and *y* are passed as a concatenated address to the next module in the pipeline. Next, a stage corresponding to the heat map is utilized. This module holds a matrix represented as a block RAM of depth *m*×*n*, since the coordinates are not sub-sampled and have the ranges 1 ≤ *x* ≤ *m* and 1 ≤ *y* ≤ *n*. For each new input address, its value in memory is incremented.

Since the aim of the detection algorithm is to calculate the average of the coordinates with the highest activation, it would be inefficient to find these event addresses after *S*_*d*_ events. Therefore, the coordinates with the highest count are stored in a FIFO element while the counting is performed. At the end, this will contain all the *x* and *y* coordinates needed for the average calculation. Once the classification flag is triggered, all the coordinates stored in the previous step (which belong to the highest activation) are acquired for calculating the total activation (the divisor). Subsequently, it will calculate the sum of the respective *x* and *y* values, and pass these as dividends to hardware dividers that will provide the final coordinates of the detected object. Algorithm 2 summarizes the above object detection hardware pipeline clearly.

**Algorithm 2:** Event-based FPGA Object Detection

**Input**: Filtered event stream $\left\{\hat{e}\right\}$, detector landmarks *l*, number of events *S* **Output**: Mean object location (*x*_*obj*_, *y*_*obj*_)
1: Initialize detector count *D*(*y, x*) = 0_*m,n*_, detector cut-off *threshold* = 0
2: **for** *t* = 1 : *S* **do**
3: For each incoming event ${\hat{e}}_{t}={\left(x_{t},y_{t},t_{t},p_{t},x_{t}^{T}\right)}^{T}$
4: For **x**_*t*_ get leaf node index *l*_*t*_ using k-d tree
5: **if** *l*_*t*_ ∈ *l* **then**
6: *D*(*y*_*t*_, *x*_*t*_) = *D*(*y*_*t*_, *x*_*t*_) + 1
7: **if** *D*(*y*_*t*_, *x*_*t*_) > *threshold* **then**
8: *threshold* = *threshold* + 1
9: Reset detector mean calculation FIFO
10: **end** **if**
11: **if** *D*(*y*_*t*_, *x*_*t*_) = *threshold* **then**
12: Push *x*_*t*_, *y*_*t*_ into the mean calculation FIFO
13: **end if**
14: **end** **if**
15: **end** **for**
16: Output the mean of the coordinates in the FIFO as (*x*_*obj*_, *y*_*obj*_)

### 2.5. Experiment Setup

**Figure 4:**
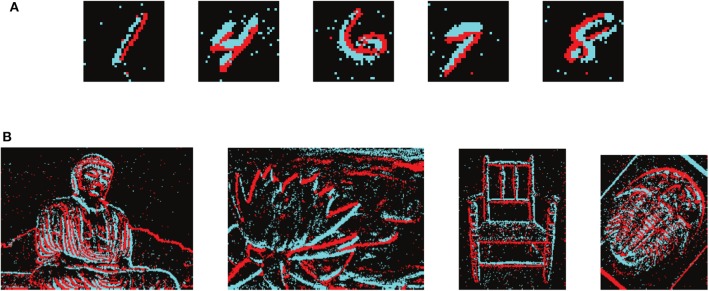
Samples from the Event-based Benchmark Datasets: **(A)** N-MNIST Samples, **(B)** N-Caltech101 Samples.

We validated our approach on two benchmark datasets (Orchard et al., 2015a), namely the N-MNIST and N-Caltech101, that have become de-facto standards for testing event-based categorization algorithms. Figure 4 shows some representative samples from N-MNIST and N-Caltech101.

**Figure 5:**
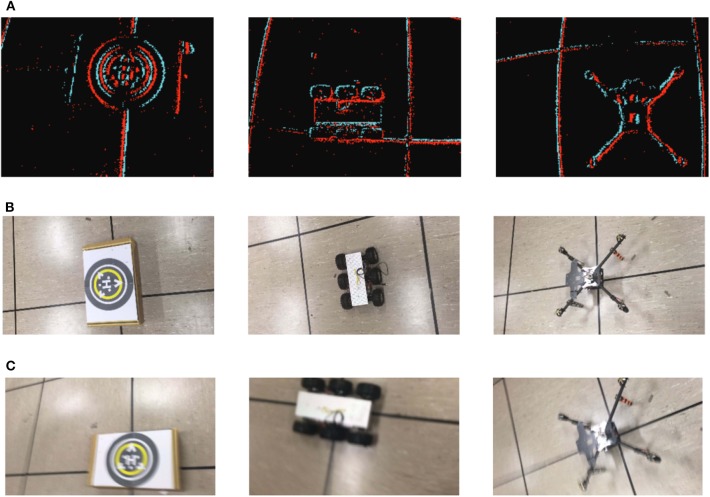
Samples from the in-house datasets containing a Landing platform, UAV and Thumper (Empty Floor as background class): **(A)** N-SOD dataset, **(B)** Frame-based dataset similar to N-SOD, **(C)** Frame-based dataset with blur similar to N-SOD.

The above datasets are good for only evaluating the categorization module. In addition, as the benchmark datasets were generated by displaying images on a monitor with limited and predefined motion of the camera, they do not generalize well to real-world situations. To overcome these limitations, we created a new dataset by directly recording objects in lab environment with a freely moving event-based sensor. The in-house dataset, called as Neuromorphic Single Object Dataset (N-SOD), contains three objects with samples of varying length in time (up to 20 s). The three objects to be recognized are a thumper 6-wheel ground robot, an unmanned aerial vehicle, a landing platform along with a background class (Figure 5A). The proposed object categorization and detection framework based on PCA-RECT is compared to state-of-the-art event-based works and thus software implementation is used with double numeric precision.

For real-time experiments, we use the commercial event camera, the Dynamic and Active-pixel Vision Sensor (DAVIS) (Brandli et al., 2014). It has 240 × 180 resolution, 130 dB dynamic range and 3 microsecond latency. The DAVIS can concurrently output a stream of events and frame-based intensity read-outs using the same pixel array. An event consists of a pixel location (*x*, *y*), a binary polarity value (*p*) for positive or negative change in log intensity and a timestamp in microseconds (*t*). In this work, polarity of the events are not considered, and only the event stream of the DAVIS is used.

#### 2.5.1. Parameter Settings

The time thresholds for the nearest neighbor filter and the refractory filter are nominally set to be Θ_*noise*_ = 5 ms and Θ_*ref*_ = 1 ms, respectively, as suggested in Padala et al. (2018). We used a FIFO buffer size of 5000 events for dynamically updating the count matrix as and when events are received. Subsequently, the RECT representation with a 2 by 2 averaging filter without zero padding at the boundaries is used to obtain a 9 × 9 feature vector for all event locations. We also experimented with other feature vector dimensions using a 3 × 3, 5 × 5, 7 × 7 sampling region and found that increasing the context improved the performance slightly. For obtaining the PCA-RECT representation, the number of PCs can be chosen automatically by retaining the PCs that hold 95% eigenenergy of the training data. For testing on the benchmark datasets, a codebook size of 3000 along with spatial pyramid matching (SPM) (Lazebnik et al., 2006) was universally used with a *k*-d tree with backtracking to find precise feature matches.

## 3. Results

### 3.1. N-MNIST and N-Caltech101

The object categorization results on the N-MNIST and N-Caltech101 datasets are given in Table 1. As it is common practice, we report the results in terms of classification accuracy. The baselines methods considered were HATS (Sironi et al., 2018), HOTS (Lagorce et al., 2016), HFirst (Orchard et al., 2015b), and Spiking Neural Network (SNN) frameworks reported in Lee et al. (2016) and Neil et al. (2016) and Gabor-SNN as reported in Sironi et al. (2018).

**Table 1:** Comparison of classification accuracy on event-based datasets (%).

	**N-MNIST**	**N-Caltech101**
H-First	71.20	5.40
HOTS	80.80	21.0
Gabor-SNN	83.70	19.60
HATS	**99.10**	64.20
vPCA-RECT (this work)	98.72	70.25
PCA-RECT (this work)	98.95	**72.30**
Phased LSTM	97.30	–
Deep SNN	98.70	–

*The bold values correspond to the best results for the N-MNIST and N-Caltech101 datasets*.

On the widely reported N-MNIST dataset, our method is as good as the best performing HATS method (Table 1). Moreover, other SNN methods are also in the same ballpark, which is due to the simple texture-less digit event streams giving distinct features for most methods. Therefore, it is a good benchmark as long as a proposed method performs in the high 90's. A test on the challenging NCaltech-101 dataset will pave way for testing the effectiveness close to a real-world scenario. Our method has the highest classification rate ever reported for an event-based classification method on the challenging N-Caltech101 dataset. The unpublished HATS work is the only comparable method in terms of accuracy, while the other learning mechanisms fail to reach good performance.

#### 3.1.1. Vary Hyper-Parameters

There are two important considerations while using the RECT representation: the feature dimension *d* obtained from the filtered matrix Equation (7) and the size of the filter itself (*p* × *q*). Another way of interpreting the feature dimension is the “square grid length” that determines the number of filtered cells (these are the opaque rectangular grids containing more than one event in Figure 1) aggregated from the filtered event count matrix. This is easier to visualize and also vary in steps of 3 × 3, 5 × 5, 7 × 7, etc. In a similar vein, the pooling of the event count matrix (*C*) using the rectangular filter *A*(*p, q*) results in a “sub-sampled” representation *R* ∈ ℝ^(*m*/*p*) × (*n*/*p*)^, and consequently, choosing a RECT patch of dimension *d* centered at *R*(*y*/*p, x*/*p*) is equivalent to choosing a “larger radius” in the event count matrix *C* ∈ ℝ^(*m*) × (*n*)^ and then performing filtering and aggregation. Again, it is easy to vary and visualize this RECT radius, say in steps of 5, instead of choosing various combinations of filter sizes (*p, q*). In the following, we vary the RECT grid and the radius to investigate the effects on classification performance using the N-Caltech101 dataset.

**Figure 6:**
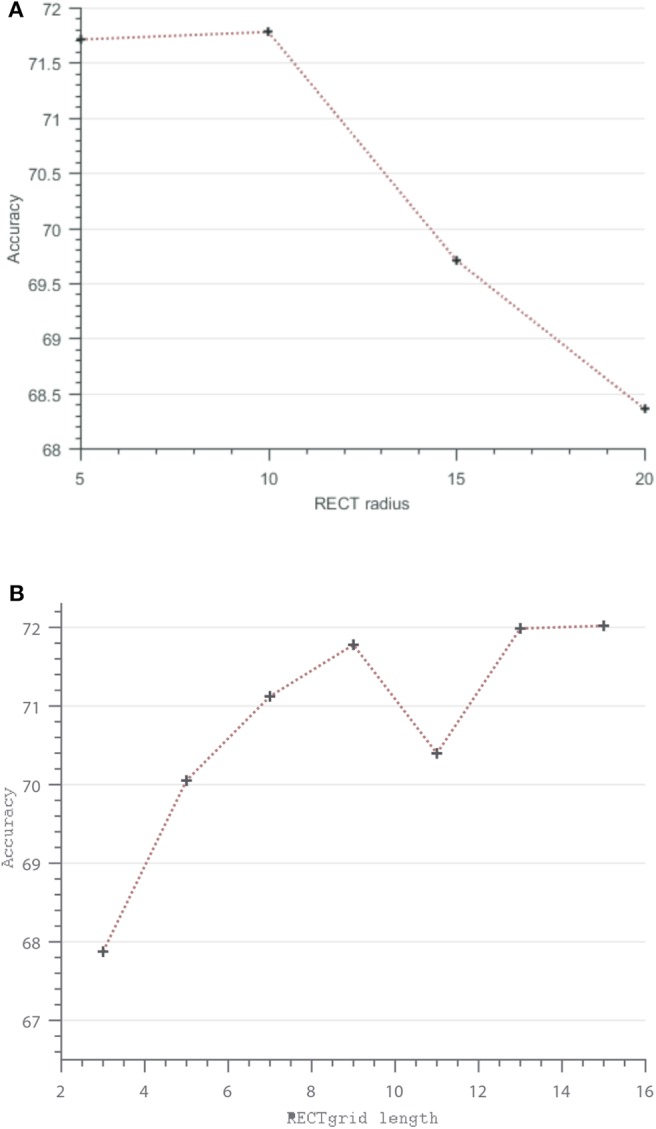
Vary RECT parameters: **(A)** RECT radius vs. accuracy, **(B)** RECT square grid length vs. accuracy.

Figure 6A illustrates the classification performance trend observed for increasing radius of the event descriptor while keeping the resolution of the grid fixed. Similar to the trend observed in Ramesh et al. (2019a), a radius of more than 10 pixels results in sub-optimal performance. On the other hand, Figure 6B shows the effect of varying grid resolution on the accuracy. It is interesting to see that as the contextual information is captured finely using denser grids, while fixing the RECT radius to 10, there is a general increase in accuracy at the expense of increase in feature dimension. For instance, a 11 × 11 grid already results in a high feature dimension of 121 and thus increasing the complexity of the subsequent feature matching step using the *k*-d tree. In our application using N-SOD, presented in the next subsection, a 9 × 9 grid with a radius of 10 was used. Next, the performance of the feature selection methods are investigated.

**Figure 7:**
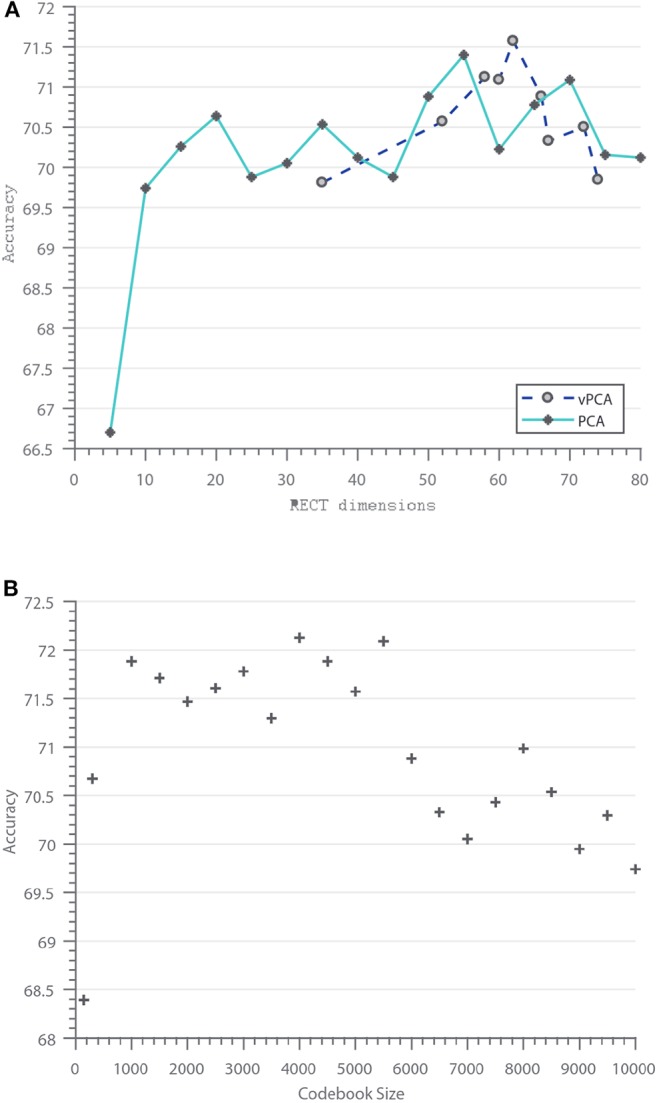
Vary feature dimension and codebook size: **(A)** Feature selection methods (PCA, vPCA) vs. Classification Accuracy, **(B)** Codebook size vs. Classification accuracy.

Figure 7A shows the performance of the feature selection methods (PCA and vPCA). As expected for PCA, increase in the number of PCs results in better performance until about the 95% eigenenergy cut-off, which is typically about 60 in our case. It is also worth noticing that just retaining five dimensions can give better performance compared to existing works. For vPCA feature selection, the number of selected features depends on the size of the smaller evaluation codebook. The smaller the evaluation codebook, the lesser the entropy, and thus lesser the number of features selected. Similar to PCA, when noisy features are discarded from the RECT representation, the classifier performance increases.

Besides feature selection, larger dictionaries or codebooks tend to provide higher classification accuracy (Nowak et al., 2006), however, the high-dimensionality of the object representation when combined with spatial pyramid matching (14 times the codebook size for a 1 × 1, 2 × 2, and 3 × 3 SPM representation) can degrade the performance for larger codebooks, as shown in Figure 7B, where two distinct clusters can be spotted. Codebook sizes less than 5000 with SPM perform better than larger codebooks. This trend has been observed in previous works as well (Lazebnik et al., 2006).

### 3.2. N-SOD

For testing on the N-SOD dataset, we divide the dataset into training and testing, with 80% temporal sequence samples per class for training and the remaining for testing. Using the training features, a dictionary is generated. Since the temporal sequences are of different length, for a fixed number of events, say every 10^5^ events, an object representation is extracted using the codebook and a linear SVM classifier is trained. Similarly for testing, for every 10^5^ events, the object representation is classified using the SVM.

Based on the above setup, an accuracy of 97.14% was obtained (Table 2) with a dictionary size of 950, which resulted in a *k*-d tree with 10 layers. We also experimented with lower dictionary sizes such as 150, 300, 450, etc., and the performance drop was insignificant (>96%). On the other hand, using a *k*-d tree with backtracking, descriptor normalization, etc., achieved close to 100% accuracy on offline high-performance PCs, which of course does not meet low-power and real-time requirements. In summary, the proposed vPCA-RECT method with a backtracking-free *k*-d tree implementation mildly compromises on accuracy to handle object detection and categorization using an event camera in real-time.

**Table 2:** Confusion Matrix (%) for the best result on the in-house N-SOD dataset.

	**Background**	**LP**	**Thumper**	**UAV**
Background	95.4128	0.3058	3.3639	0.9174
LandingPlatform	0	99.2268	0.5155	0.2577
Thumper	0	1.9257	96.9739	1.1004
UAV	0	0	3.1884	96.8116

We report the precision and recall of the detection results by ascertaining if the mean position of the detected result is within the ground truth bounding box. We obtained: (a) *Precision*- (498/727) = 0.685: The percentage of the detections belonging to the object that overlap with the groundtruth (b) *Recall*- (498/729) = 0.683: The percentage of correct detections that are retrieved by the system. The number of “landmarks" were set to 20 in the above experiments while similar results were obtained for values such as five and ten. It is worth pointing out that the codebook size used for 4-class N-SOD detection and recognition, thereby for the FPGA implementation, need not be in the thousands as with the complex N-Caltech101 dataset for giving high accuracy.

### 3.3. FPGA Performance

The hardware implementation and performance of the Xilinx Zynq-7020 FPGA running at 100 MHz was evaluated by direct comparison with the results of the algorithm's software version in MATLAB. The Zynq was interfaced to a down-looking DAVIS camera, on-board an unmanned aerial vehicle flying under unconstrained lighting scenarios. We recommend viewing our submitted video^1^ that clearly shows the classification/detection process better than still images. Vivado design suite was used for synthesis and analysis of the design. The in-built logic simulator ISIM was used for testing; first, to verify that the behavior was met, and later for verification of timing performance and latency requirements post-synthesis and post-implementation.

#### 3.3.1. Timing

The time taken for a single event to be classified for the worst possible *k*-d tree path was 560 nanoseconds, where roughly 80% of the time is employed traversing the tree. The rest is employed for buffering (5%), count matrix updating (5%), and SVM inference (10%). On the other hand, the detection task includes a comparison of codewords and consequent updating of the detection count matrix, which happens for each event and takes 50 nanoseconds. Later, the mean calculation between the respective coordinates consists of a summation and division. The former is proportional to the number of values in the operation and takes one clock cycle per element (in operation this approximates to 5 values), and the latter requires 80 nanoseconds of processing. This amounts to 130 nanoseconds which is negligible since it only happens once for every set of valid classified events (*S*_*d*_).

Due to the asynchronous nature of the sensor, it is not uncommon to receive a consecutive batch of events in a very short period (say 10 μs). These events cannot be handled in parallel, since each of them modifies the classification count matrix, and the SVM feature representation. Then, the events that arrive while the tree is been traversed are buffered and later processed. This may add a delay in the output of about 2 event cycles (about 1 μs) depending on the amount of events triggered at the same instant, however, the refractory filter avoids this case for multiple events triggered at the same pixel. In any case, the DAVIS camera output has a minimum event throughput at 1μs (mean inter-event interval about 10 μs), and thus is a rare case inhibiting real-time processing.

The classification and detection tasks are performed in parallel and follow different periods of operation. Classification is applied on a FIFO storing the last *S*_*c*_ events, and it is consequently updated for each incoming event. Hence, a classification output is provided for each new input data. Separately, the detection pipeline works on a periodic basis and only for a specific classification result, providing a valid output once every *S*_*d*_ events.

The latency of the system is on par with similar works on neuromorphic vision tracking on FPGA (Moeys et al., 2016; Linares-Barranco et al., 2019), taking into consideration that these works are implemented for low-level object tracking. Additionally, our system outperforms similar applications using frame-based cameras using FPGA or microprocessors, which by definition normally operate in the scale of milliseconds. **Table 4** presents a summary of these measurements.

#### 3.3.2. Resource Utilization

A summary of utilization of hardware elements can be seen in Table 3. The modules corresponding to the *k*-d tree and SVM require memory initialization to store tree nodes properties and SVM coefficients. Hence, Read-Only Memory (ROM) was utilized for this purpose. This accounts to 128 KB for the *k*-d tree module and 180 KB for the SVM module. These resources are synthesized into RAM blocks in the FPGA, but these are only used for reading as would be the case with a regular ROM element. Digital signal processing (DSP) slices were utilized to perform integer division. There are two division operations in the detection pipeline, and each of these dividers require two DSP slices; one multiply block and one multiply adder block.

**Table 3:** Hardware utilization report for the FPGA running the proposed modules.

	**Utilization**	**Available**	**Utilization %**
LUT	18238	53200	34.28
LUTRAM	12124	17400	69.68
FF	2065	106400	1.94
BRAM	48	140	34.29
DSP	4	220	1.82
IO	102	200	51.00

#### 3.3.3. Power Consumption

Table 4 also lists the power consumption of the vPCA-RECT system in comparison to state-of-the-art methods. For our system, the DAVIS event camera operates at a few milliwatts (10 mW) while the Zynq operates at about 3 W including the base power for running Ubuntu. The algorithmic implementation itself increases the dynamic on-chip power by only 0.37 W. As a comparison, event-based blob tracking implementation on FPGA (Moeys et al., 2016) reported 0.775 W running at 50 MHz. In general, FPGA-based recognition systems for RGB cameras (Schlessman et al., 2006; Hikawa and Kaida, 2015; Mousouliotis et al., 2018), which present solutions running at equal or lower clock frequencies, consume more power than our implementation. Similarly, Zhai et al. (2013) and Ali et al. (2009) present works that take advantage of the mixed computation capabilities of the Xilinx Zynq chip, but get hindered by the high latency characteristic of a frame-based system.

**Table 4:** Comparison of power consumption and latency of existing object detection systems with the proposed method.

	**Frequency (MHz)**	**Power (Watts)**	**Latency (ns)**
vPCA-RECT (ours)	100	**0.37**	560
Moeys et al. (2016)	50	0.78	440
Zhai et al. (2013)	58	0.90	11000 k
Ali et al. (2009)	50	0.66	91000 k
De Smedt et al. (2015)	2600	22.0	~

*The bold value correspond to our system's power consumption*.

To provide a broader context to the power consumption of frame-based systems, let us consider (De Smedt et al., 2015) (Brix embedded system, Intel-I7 processor with 8G RAM) used for real-time object tracking. It consumes about 22 W, which is 7x more than our full hardware implementation. Note that the Zynq module is a powerful and flexible development tool, but far exceeds the utilities compared to SmartFusion FPGAs that allow sleep modes, non-volatile configuration memory, and have much lower power consumption overall. In other words, there is significant room for very low-power implementation (less than 1 W) of our framework with appropriate hardware choices and development efforts.

### 3.4. Comparison to CNN

**Figure 8:**
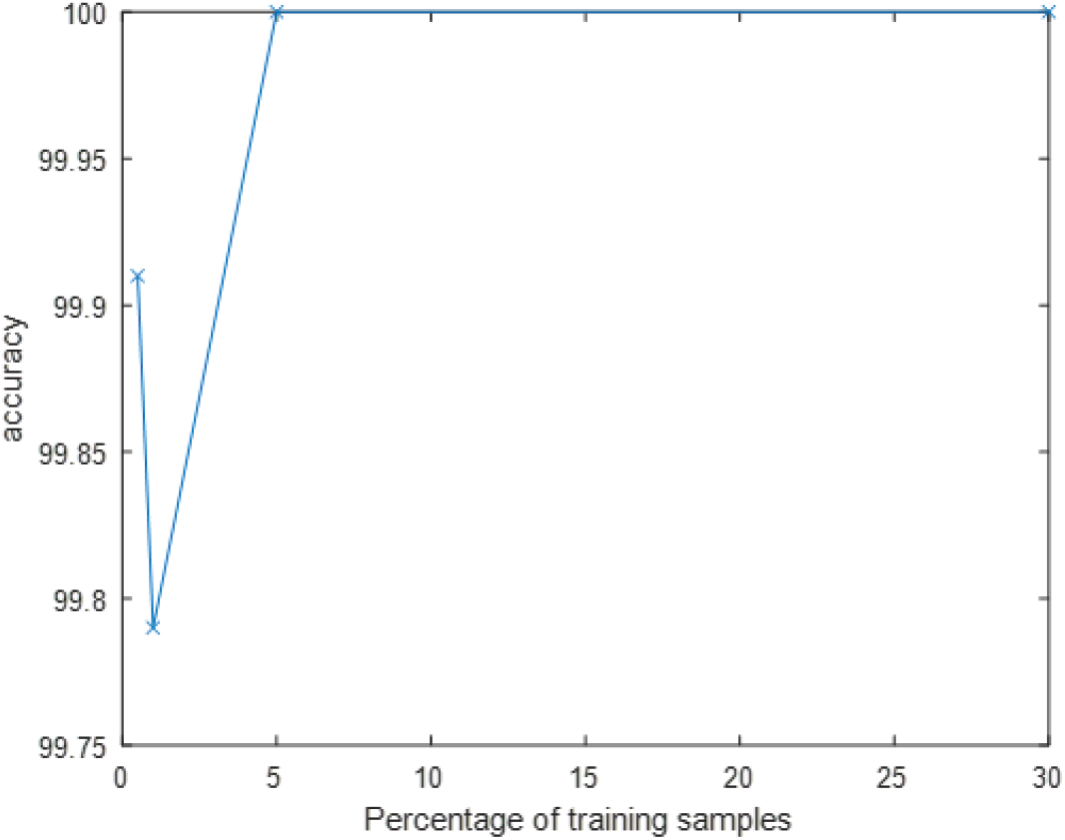
Alexnet test accuracy vs. Percentage of training samples.

In order to compare to state-of-the-art deep neural networks, we recorded a similar dataset to N-SOD using a frame-based camera (Figure 5B) and transfer learning via AlexNet classified the object images. The total number of images recorded were in the order of 6000. With an equivalent train/test split compared to N-SOD, perfect performance can be achieved on the clearly captured test images. In fact, as Figure 8 shows, perfect performance can be achieved on the test images with as little as 5% of the data used for training. It is indeed surprising to see that with 0.5% training data (8 samples per object category), the accuracy can be near perfect.

However, when we tested the Alexnet model (trained on normal images) on a dataset under motion blur conditions (Figure 5C), an accuracy of only 79.20% was obtained. It was clear that the black UAV frame when blurred looks like the black-stripped background and creates much confusion as seen from Table 5. This confirms the disadvantage of using frame-based cameras to handle unconstrained camera motion. Note that fast camera motion leads to only an increase in data-rate for event-based cameras and has no effect on the output. In fact, recordings of N-SOD have significant amount of such fast motions.

**Table 5:** Confusion Matrix (%) for CNN classifier on the “blur” frame-based dataset (%).

	**Background**	**LP**	**Thumper**	**UAV**
Background	100.00	0	0	0
Landing platform	0.64	99.36	0	0
Thumper	9.63	0	90.37	0
UAV	72.02	0	9.3	27.05

Additionally, we recorded images to test the performance of CNN on data captured under low-lighting conditions and slow motion conditions. A near-perfect performance on these set of images was impressive, as the features extracted by CNN were robust enough to be invariant under extreme lighting conditions. Similarly, one could argue that “blurred” images when included in the training will boost the accuracy of the deep learning model. We confirmed that by training on 30% normal (1976 images) plus 3% blur (72 images), and testing on the rest of the data captured under normal, blur, and low-lighting conditions. This mixed testing allowed the CNN to correctly classify the UAV blurred images (99.4% accuracy). Nonetheless, this is a rather unnatural training setting, one that is not expected to be deployed in the real-world. Moreover, other works have also concluded that existing networks are indeed susceptible to many image quality issues, particularly to blur and noise (Dodge and Karam, 2016).

## 4. Discussion

We have demonstrated object detection and categorization in an energy-efficient manner using event cameras, where the only information that is important for these tasks is how edges move, and the event camera naturally outputs it. The proposed PCA-RECT feature takes advantage of this sparsity to generate a low-dimensional representation. The low-dimensional representation is further exploited for feature matching using a *k*-d tree approach, capable of obtaining the best performance on the challenging Neuromorphic Caltech-101 dataset compared to state-of-the-art works.

Although *k*-d trees enable fast and large scale nearest neighbor queries among high dimensional data points, such as those produced by RECT or PCA-RECT^2^, their application is restricted to efficiently computing distance measures. Thus, as long as there are descriptors, global or local, *k*-d trees are a good fit to both event data and RGB frames. Nonetheless, it remains to be seen whether global event-based descriptors, say HATS (Sironi et al., 2018), will benefit from *k*-d trees. On the other hand, the sparsity of events leads to less data compared to intensity frames recorded at 30 Hz or 10 MB/s (the DAVIS outputs typically at 150KB/s). This tends to lend well to the use of *k*-d trees, given there will be lesser information to build and decode. Overall, *k*-d trees could be better utilized for real-time and embedded applications for event camera data compared to RGB frames, and its performance remains to be fully explored.

It is important to note that we demonstrated very competitive performance compared to Deep SNN on the N-MNIST dataset using the proposed dictionary-based framework. However, it is indeed expected that deep features learned using neural networks shall outperform hand-crafted features, such as PCA-RECT, in the future. Even so, it is non-trivial as to how a deep learning approach can be effectively and efficiently suited to a purely spike-based or event-based data. In this work, real-time FPGA implementation was achieved with several careful design considerations, such as a backtracking-free *k*-d tree for matching to the codewords, a virtual PCA-RECT representation obtained by analyzing the *k*-d tree partitioning of the feature space, etc. To the best of our knowledge, this is the first work implementing a generic object recognition framework for event cameras on an FPGA device, verified in a lab demo setting under unconstrained motion and lighting setup, thereby demonstrating a high performance over resource ratio. Additionally, it is well-known that dictionary-based methods easily scale to the number of samples, since performance depends only on the codebook size. For instance, searching a large image dataset with 10 million images takes only about 50 ms (Jégou et al., 2010) using compact representations of descriptors. This type of large-scale recognition using event cameras shall be a future research direction for us and the larger neuromorphic vision community.

In terms of comparison to a frame-based setup, we found the average elapsed time for feature extraction of a single image under CPU execution environment (0.6726 s) hindered real-time performance. However, this latency can be drastically reduced using GPUs while power consumption increases significantly. This is an important case where frame-based paradigm is unfavorable compared to the low-power event-based implementation presented in this paper. Additionally, under fast moving conditions of the sensor, frame-based CNN was shown to perform unreliably. Thus, when event-based processing can accomplish higher accuracy and reliability under low-power settings, as demonstrated in this work under closed-loop conditions, there is great potential for silicon retinas as an alternative or complimentary visual sensor for myriad other applications.

## Data Availability Statement

All datasets generated for this study are included in the article/supplementary material. The N-SOD data set is available at https://tinyurl.com/s84nlm4 and the source code (MATLAB and VHDL) is available at https://github.com/nusneuromorphic.

## Author Contributions

BR: thesis director and main contributor. Formalized the theory, implemented the algo in MATLAB and evaluated the results. AU: ported the algo to FPGA hardware and evaluated the results. HY: assist BR in software experiments and verify hardware feasibility using CPP implementation. LD: hardware system integration and filtering implementation. GO: co-supervisor and instigator of the work.

### Conflict of Interest

The authors declare that the research was conducted in the absence of any commercial or financial relationships that could be construed as a potential conflict of interest.

## Footnotes

^1^Demo: https://youtu.be/yWfCmHnV5f0

^2^PCA-RECT feature can be high-dimensional when the number of chosen dimensions are close to that of the corresponding RECT representation.
